# Synthesis, Characterization, and Stability of Two Americium Vanadates, AmVO_3_ and AmVO_4_

**DOI:** 10.1021/acs.inorgchem.3c00251

**Published:** 2023-06-05

**Authors:** Jean-François Vigier, Thierry Wiss, Natalia Palina, Tonya Vitova, Jean-Yves Colle, Daniel Bouëxière, Daniel Freis, Rudy J. M. Konings, Karin Popa

**Affiliations:** † European Commission, Joint Research Centre (JRC), Karlsruhe 76125, Germany; ‡ Institute for Nuclear Waste Disposal (INE), Karlsruhe Institute of Technology, P.O. 3640, D-76021 Karlsruhe, Germany

## Abstract

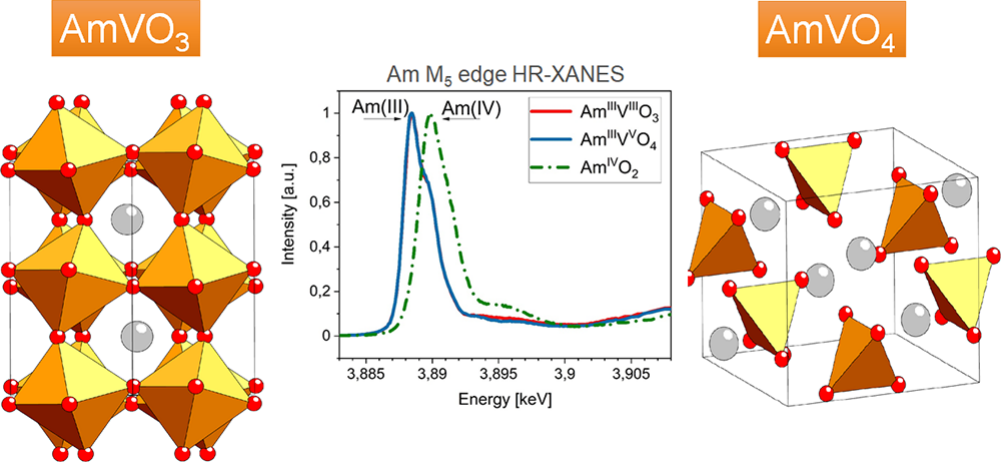

In search for chemically stable americium compounds with high power densities for radioisotope sources for space applications, AmVO_3_ and AmVO_4_ were prepared by a solid-state reaction. We present here their crystal structure at room temperature solved by powder X-ray diffraction combined with Rietveld refinement. Their thermal and self-irradiation stabilities have been studied. The oxidation states of americium were confirmed by the Am M_5_ edge high-resolution X-ray absorption near-edge structure (HR-XANES) technique. Such ceramics are investigated as potential power sources for space applications like radioisotope thermoelectric generators, and they have to endure extreme conditions including vacuum, high or low temperatures, and internal irradiation. Thus, their stability under self-irradiation and heat treatment in inert and oxidizing atmospheres was tested and discussed relative to other compounds with a high content of americium.

## Abstract 2

AmVO_3_ and AmVO_4_ were prepared by a solid-state reaction and characterized in terms of crystal structure, americium and vanadium oxidation states, thermal behavior, and self-irradiation stability. With a fast amorphization behavior, large volume variation, and interchangeable structure as a function of the presence of the oxygen atmosphere and temperature, americium vanadates do not appear to be good candidates as Am forms for radioisotope power systems.

## 1 Introduction

The americium isotope ^241^Am is formed during storage of plutonium via β^–^-decay of ^241^Pu with a half-life of 14.33 years. Due to ^241^Am accumulation in existing stocks of civil-separated plutonium in Europe, and its relatively high specific power of 0.114 W/g, ^241^Am has been proposed for use in radioisotope power systems (RPSs)^1^ and is under consideration by the European Space Agency (ESA) as an energy source for future European space missions.^2^ The requirements for a stable solid form are very diverse, ranging from storage on earth and operation in space to safe performance in case of accidents and post-accident scenarios. Our group studied several ceramic forms containing significant specific Am amounts,^3−5^ the present study being in line with the past efforts.

The synthesis and crystal structure of AmVO_3_ were reported for the first time by Keller and co-workers.^6,7^ In their work, AmVO_4_ was prepared by dissolving AmO_2_ and V_2_O_3_ as hydroxides, mixed, dried, and reacted at 1250 °C under an oxidizing atmosphere. The produced AmVO_4_ was subsequently reduced to obtain AmVO_3_. The authors proposed the crystal structure for the two compounds (perovskite-type for AmVO_3_ and zircon-like for AmVO_4_) and gave the lattice parameters.

According to the Inorganic Crystal Structure Database (ICSD), there are no compounds of americium and vanadium with a fully characterized crystal structure. We present here the complete crystal structure refinement of the two vanadates by Rietveld analysis at room temperature. Spectroscopic and microscopic studies were carried out to check the purity of the samples. The high-resolution X-ray absorption near-edge structure (HR-XANES) technique was applied at the Am M_5_ edge to characterize the Am oxidation states. The stability of AmVO_3_ and AmVO_4_ under self-irradiation and heat treatment in an inert and oxidizing atmosphere was tested and discussed relative to other americium-containing compounds.

## 2 Experimental Section

Caution: ^241^Am is a highly radioactive isotope (*t*_1/2_ = 432.8 years, specific activity of 126.8 GBq/g). All work presented in this paper were carried out in glove boxes in radiological laboratories licensed for handling actinides. When appropriate, shielding and remote handling tools were used to protect the workers in these experiments.

### 2.1 Sample Preparation

#### 2.1.1 AmVO_3_

Stoichiometric amounts of ^241^AmO_2_ (158 mg of aged material containing about 7% ^237^Np as the decay product and 2% ^239^Pu) and V_2_O_5_ (48 mg, Merck, 99.95%) were mixed in an agate mortar and pressed into a pellet. The thermal treatment was performed for 10 h at 1250 °C under Ar/H_2_ (5%) with heating and cooling ramps of 200 °C/h.

#### 2.1.2 AmVO_4_

A chip of 78 mg of the previous AmVO_3_ pellet was oxidized for 2 h up to 1000 °C under oxygen by using a Netzsch STA 449C thermogravimetric analysis instrument. Oxidation started at about 400 °C with a maximum oxidation rate at 450 °C, as discussed in the Materials Characterization section. This synthesis was done independently from the thermogravimetric analysis described below during in which higher temperatures were reached.

### 2.2 Thermogravimetric Analysis

The thermal behaviors of AmVO_3_ and AmVO_4_ were assessed under an argon/hydrogen and oxygen atmosphere, respectively (to preserve the oxidation state of the respective compounds) using a Netzsch STA 449C thermogravimetric analysis instrument. The temperature was controlled by a Pt-PtRh (10%) thermocouple. The measurements were conducted on pellet fragments (11–78 mg) up to 1500 °C in alumina crucibles, and the applied heating and cooling rates were 10 °C/min.

### 2.3 XRD

Room temperature XRD analyses were performed on about 10 mg of powdered material loaded in a bicomponent epoxy resin using a Bruker D8 Advance diffractometer (Cu Kα radiation, 40 kV, 40 mA) mounted in a Bragg–Brentano configuration. The diffractometer was equipped with a curved Ge(1,1,1) Kα_1_ monochromator, a ceramic copper tube, and a LinxEye position sensitive detector. The XRD patterns were recorded using a step size of 0.01° across the 10° ≤ 2θ ≤ 120° angular range. Structural analysis was performed by the Rietveld method using the Jana2006 software.^8^

Since all the crystallographic parameters could not be independently refined, some structural constrains were applied. For the atomic displacement parameter, isotropic displacement parameters *U*_iso_ were used, and the following equation has been considered:^9^
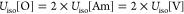
1

The V–O distances were constrained to 2 Å for the perovskite structure AmVO_3_^10^ and to 1.71 Å for the zircon structure AmVO_4_.^11^

### 2.4 Raman Spectroscopy

Raman spectroscopy measurements were performed on fragment of pellets (2–3 mg) at room temperature and ambient pressure on a polycrystalline specimen using a Horiba Jobin-Yvon T64000 spectrometer. For technical reasons, two lasers at different wavelengths were used; a 647 nm Kr+ laser and a 660 nm solid state laser excitation source. A 50× long focal objective was used to irradiate the sample and collect the back-scattered light. Great care was taken to avoid sample damage or laser-induced heating. Measurements were performed with few tenths of a milliwatt incident power.

### 2.5 Scanning Electron Microscopy

Images of the samples (fragment of pellets of 1–2 mg) were obtained in a Philips/FEI XL40 SEM operated at 25 kV, equipped with a SAMx energy-dispersive X-ray analysis system (EDX). This microscope (high-voltage unit, column, chamber, and turbomolecular pump) was placed inside a glovebox, while the components that are not getting in contact with the active materials (primary vacuum system, the water-cooling circuit, and the acquisition electronic) were outside.^12^

### 2.6 XANES and HR-XANES

For AmVO_3_ and AmVO_4_ compounds, the Am M_5_ HR-XANES spectroscopy technique was performed at the ACT station of the beamline for catalysis and actinide research (hereafter CAT-ACT beamline) of the KIT Light Source, Karlsruhe, Germany.^13^ Spectra acquisitions were done utilizing a Johann type X-ray emission spectrometer. The incident beam was monochromatized by a Si(111) double-crystal monochromator (DCM), focused to 500 × 500 μm, and subsequently narrowed down by slits onto the sample to a spot size of about 200 × 200 μm. The X-ray emission spectrometer consists of four Si(220) crystals with a 1 m bending radius and a single diode VITUS silicon drift detector (Ketek, Germany), which together with the sample were arranged in a Rowland circle geometry.^13^ AmO_2_ reference sample was used to calibrate Am M_5_ HR-XANES spectra. The main absorption maximum was set to 3890 eV for AmO_2_.^14^ The maxima of the WLs are located at AmCl_6_^3–^ (3888.4 eV), AmFe_2_ (3887.5 eV) reported elsewhere.^15,16^ Both Am M_5_ edge data cited above were measured in conventional fluorescence mode and were similar to AmVO_3_ and AmVO_4_ (3888.5 eV) recorded in this work. The slight divergence of the absorption maximum of AmO_2_ in the current work (3890 eV) from the data reported by Epifano *et al.*(17) (about 3891.5 eV) is a result of different experimental energy resolutions (for details cf. Discussion in the Supporting Information and Figure S5). The multi-position sample cell (containing <1 mg powdered material embedded in bicomponent glue) was placed into a double containment, where the inner compartment was sealed by 8 μm and the outer compartment by 13 μm Kapton foil. The HR-XANES spectra were measured with a step size of 0.1 eV from −10 to +25 eV from the white line (WL) of the respective edge and 0.5 eV in all other parts of the spectra. At least two spectra were averaged for each sample. The sample, crystals, and detector were enclosed in a box filled with helium to minimize intensity losses due to scattering and absorption of photons in air. Constant helium flow was maintained to keep the oxygen level below 0.1%.

Additionally, Am L_3_ edge and V K-edge XANES measurements were performed at the INE-Beamline^18^ of the KIT Light Source, Karlsruhe, Germany. The radiation protection measures were kept identical to those used at the ACT station.^19^ Two Ge(422) and two Si(111) crystals were mounted in the double-crystal monochromator (DCM) for Am L_3_ and V K edge measurements, respectively. The beam was focused on a ∼0.5 mm × 0.5 mm spot on the sample. Zr or V metal foils were used for energy calibration for the Am L_3_ edge or V K edge XANES, respectively. V_2_O_3_ (Sigma-Aldrich, 99.99%) and V_2_O_5_ (Merck, 99.95%) powders were mixed with cellulose and pressed into the pellets and used as references for V K edge XANES. The XANES spectra were measured in fluorescence mode with step sizes of 0.25 and 0.8 eV from −10 to +25 eV from the white line (WL) of the respective edges for the V K edge and Am L_3_ edge and 4 eV steps in the post-edge area of the spectra. At least two spectra were averaged for each sample. Measurements were performed in air, and no radiation damage was observed during the measurements.

### 2.7 α Self-Irradiation

Due to the high alpha activity of americium, the doses accumulated by the AmVO_3_ and AmVO_4_ at the time of different measurements must be considered. Daily doses of about 7.8 × 10^15^ and 7.4 × 10^15^ α/g were built up for AmVO_3_ and AmVO_4_, respectively. Only TGA and XRD techniques could be applied on freshly prepared material. The calculated doses of the materials at the time of the different measurements are summarized in Table 1. Between measurements, the samples were stored under the atmosphere of the glovebox where the samples were produced (nitrogen with up to 10,000 ppm oxygen).

**Table 1:** α Dose Accumulated in AmVO_3_ and AmVO_4_ at the Time of the Different Measurements, in α Decay Events per Gram of Material

	AmVO_3_		AmVO_4_	
	sample age, days	cumulated dose, α/g	sample age, days	cumulated dose, α/g
XRD	1.6	1.2 × 10^16^	2.7	2.1 × 10^16^
	132	1.0 × 10^18^	131	1.0 × 10^18^
	212	1.6 × 10^18^	211	1.6 × 10^18^
TGA	3.4	2.7 × 10^16^	2.5	1.9 × 10^16^
Raman	451	3.5 × 10^18^	450	3.5 × 10^18^
	890	6.8 × 10^18^	890	6.8 × 10^18^
SEM	660	5.1 × 10^18^	659	5.1 × 10^18^
Am L_3_ edge XANES, Am M_5_ edge HR-XANES	603	4.7 × 10^18^	607	4.7 × 10^18^
V XANES	726	5.6 × 10^18^	727	5.6 × 10^18^

## 3 Results and Discussion

### 3.1 Materials Characterization: Fresh Material

During the synthesis of AmVO_4_ by oxidation of an AmVO_3_ specimen, the DTA measurement (Figure S1) performed in oxygen indicated a single exothermic event reaching a maximum energy release at 450 °C with an associated weight gain of 4.5 wt %, which was attributed to the oxidation to the AmVO_4_ form (expected gain of 4.7 wt %).

**Figure 1:**
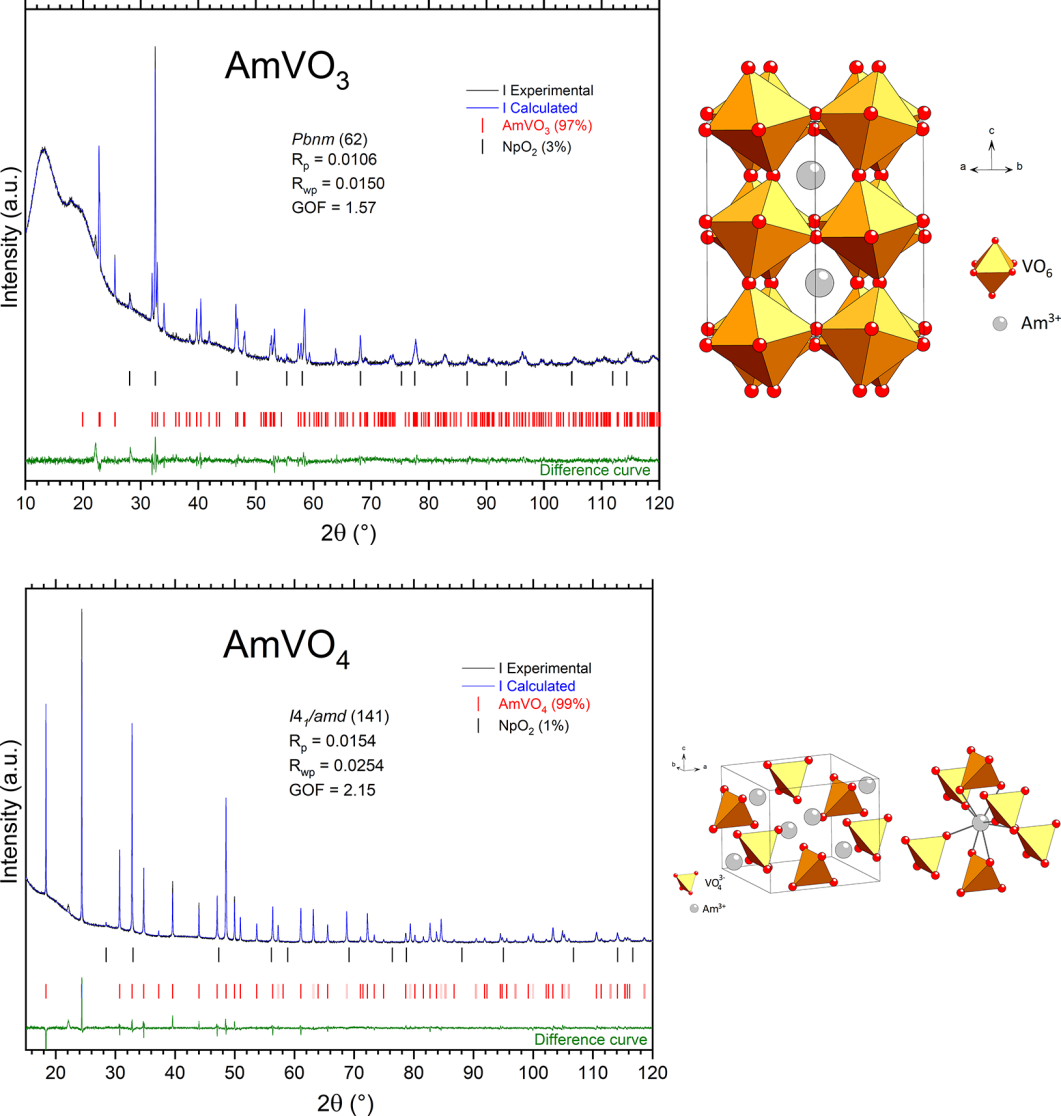
XRD patterns, Rietveld refinement, and crystal structure of AmVO_3_ and AmVO_4_.

Americium vanadate AmVO_3_ crystalizes in the perovskite *Pbnm* structure after sintering under an Ar/H_2_ atmosphere; meanwhile, after thermal treatment in oxidative conditions, the material converts into AmVO_4_ showing a zircon-like *I*4_1_/*amd* structure (Figure 1 and Table 2). Residual NpO_2_ can be detected in quantifiable amounts, which indicates that this daughter element of ^241^Am does not fully integrate the two vanadate compounds and segregates (at least partially) into an oxide phase. The Np L_3_ edge XANES data are shown in Figure S4, supporting the above statement.

**Table 2:** Unit Cell Parameters and Results of the Rietveld Refinement for AmVO_3_ and AmVO_4_^a^

formula unit	AmVO_3_	AmVO_4_	AmVO_4_ (Keller *et al.*(7))	AmVO_4_ (Goubard *et al.*(20))
space group	*Pbnm*	*I*4_1_/*amd*	*I*4_1_/*amd*	*I*4_1_/*amd*
space group number	62	141	141	141
*a*, Å	5.4445(3)	7.2938(2)	7.31(1)	7.311(2)
*b*, Å	5.5914(3)	7.2938(2)	7.31(1)	7.311(2)
*c*, Å	7.7556(4)	6.4284(2)	6.42(1)	6.422(2)
α, °	90	90	90	90
β, °	90	90	90	90
γ, °	90	90	90	90
*V*, Å^3^	236.10(2)	341.992(10)	343	343.26
*Z*	4	4	4	4
*M*, g/mol	340.00	356.00	356.00	356.00
crystallographic density, g/cm^3^	9.565	6.9142	6.89	6.89
GOF	1.57	2.15		
*R*_P_	0.0106	0.0154		
*R*_wp_	0.0150	0.0254		
*R*_exp_	0.0068	0.0072		

a *R*_p_ = Σ(*i*)[|*yi*(obs) – *yi*(calc)|]/Σ(*i*)[*yi*(obs)]; *R*_wp_ = {Σ(*i*)[*wi*(*yi*(obs) – *yi*(calc))^2^]/Σ(*i*)[*wi* × *yi*(obs)^2^]}^1/2^; GOF = *R*_p_/*R*_exp_; *R*_exp_ = {Σ(*i*)[*wi* × *yi*(obs)^2^]/(*n* – *p*)}^1/2^.

The crystal structures suggest that americium stays in the oxidation state III in the materials whatever the redox condition, while vanadium can have the oxidation state III or V, which results in *AB*O_3_ perovskite or *AB*O_4_ zircon structures, respectively. One can notice that during the oxidation process, the density of the material strongly decreases from 9.565 g/cm^3^ for the close-packed AmVO_3_ to 6.914 g/cm^3^ for the oxyanionic AmVO_4_, which indicates a volume increase of 45% during oxidation. The formation of a zircon-like structure for AmVO_4_ is in accordance with the works of Keller *et al.*(6,7) or of Goubard *et al.*(20) showing slightly lower but similar lattice parameters (Table 2).

TGA analyses, under air and argon/hydrogen for AmVO_4_ and AmVO_3_, respectively, revealed no significant weight loss for both materials, indicating a good thermal stability of the compounds at high temperature. However, XRD analysis of AmVO_3_ after TGA measurement showed the presence of a new unidentified phase, AmVO_3_ remaining as the main compound, suggesting the beginning of a degradation of the material at this temperature. In contrast, XRD measurement of AmVO_4_ after TGA showed that the material remains fully unchanged, indicating an excellent thermal stability of this material under oxidative conditions.

**Figure 2:**
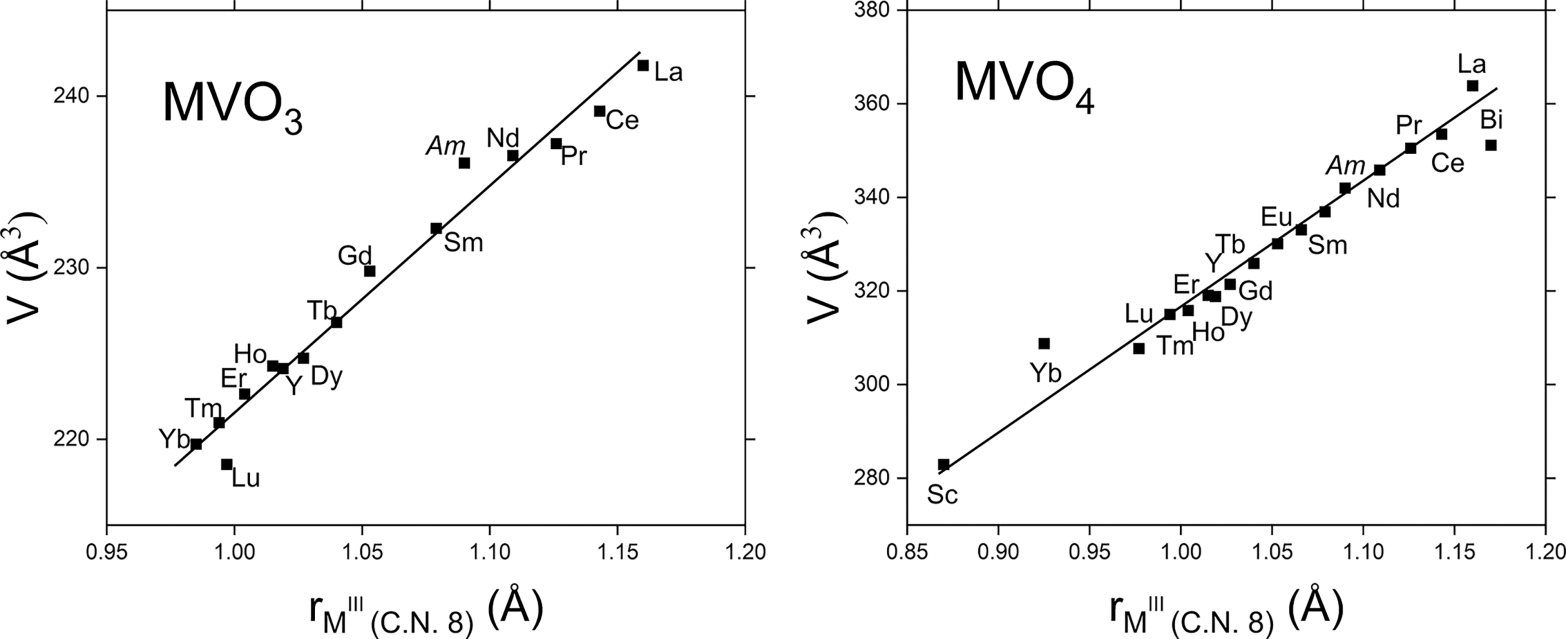
Evolution of the unit-cell volumes of MVO_3_ and MVO_4_ compounds as a function of the cationic radii. The unit-cell volume values are taken from the Inorganic Crystal Structure Database (https://icsd.fiz-karlsruhe.de) and the ionic radii from Shannon.^21^

The evolution of the unit-cell parameters/volumes of the *M*VO_3_ and *M*VO_4_ compounds (Figure 2) confirms that the Am-vanadates belong to the corresponding crystallographic families. Only the perovskite phases crystalizing in the orthorhombic *Pbnm* space group are considered in the *M*VO_3_ part of Figure 2. The atomic coordinates and displacement parameters for AmVO_3_ and AmVO_4_ compounds are presented in Table 3.

**Table 3:** Atomic Coordinates and Displacement Parameters for AmVO_3_ and AmVO_4_ Compounds

element	label	oxidation state	SOF	Wyckoff position	*x*	*y*	*z*	*U*_iso_, Å^2^
AmVO_3_ (*Pbnm*, perovskite)								
Am	Am1	+3	1	4c	0.9888(15)	0.0507(5)	1/4	0.004(1)
V	V1	+3	1	4b	1/2	0	0	0.004(1)
O	O1	–2	1	4c	0.397(8)	–0.010(9)	1/4	0.008(2)
O	O2	–2	1	8d	0.745(12)	0.263(11)	0.037(6)	0.008(2)
AmVO_4_ (*I*4_1_/*amd*, zircon)								
Am	Am1	+3	1	4a	0	3/4	1/8	0.0074(6)
V	V1	+5	1	4b	0	1/4	3/8	0.0074(6)
O	O1	–2	1	16h	0	0.4377(11)	0.2156(17)	0.0148(12)

### 3.2 Materials Characterization: α Self-Irradiated Material

Due to the impact of the COVID-19 pandemic, SEM, XANES, and Raman characterization could only be performed about 1–2 years after synthesis. Due to the high alpha activity of ^241^Am, the effect of self-irradiation must be considered in this condition, the main effect being amorphization of the crystal structure. The amorphization of the two vanadates was followed through XRD analyses, confirming that SEM, XANES, and Raman were performed on amorphous material. However, the results further described below seem to show only a limited impact of this amorphization on the microstructure (SEM) and electronic structure (XANES) of the two vanadates, while Raman spectroscopy showed limited impact of self-irradiation for AmVO_4_ and a chemical transformation due to Raman laser heating for AmVO_3_.

**Figure 3:**
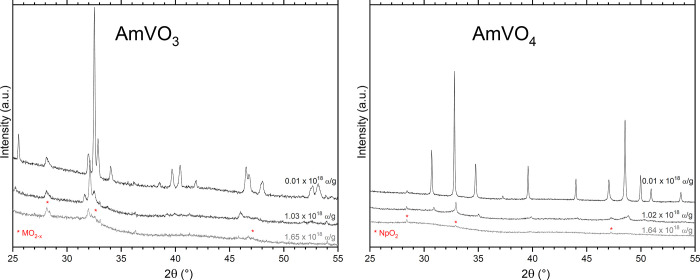
Evolution of the XRD pattern of AmVO_3_ and AmVO_4_ over time showing the progressive amorphization of the material.

The XRD results reveal that the volume of the close-packed AmVO_3_ perovskite structure increases by about 4.2% after a dose of 1.03 × 10^18^ α/g, while that of the oxyanionic AmVO_4_ zircon structure shows a contraction of about 1.8% after a similar dose (Table 4). Therefore, the increased lattice disorder under α self-irradiation in this low-density structure results in a more compact arrangement. One can clearly see the swelling in AmVO_3_ and the contraction in AmVO_4_ in Figure 3, with a shift of the diffraction peaks into lower and higher 2Θ values, respectively. After a dose of about 1.65 × 10^18^ α/g (7 months), the main diffraction peak of AmVO_3_ is still visible, which indicates that the amorphization process is well advanced but not completed, while at that dose, the amorphization of AmVO_4_ seems fully completed.

**Table 4:** Lattice Parameter Variation of AmVO_3_ and AmVO_4_ under Self-Irradiation

*a*, Å	*b*, Å	*c*, Å	*V*, Å^3^	volume variation, %	dose (α/g)	dpa^a^
AmVO_3_ (*Pbnm*, perovskite)						
5.444	5.590	7.755	235.982		0.01 × 10^18^	0.0016
5.513	5.661	7.882	245.994	4.2	1.03 × 10^18^	0.158
AmVO_4_ (*I*4_1_/*amd*, zircon)						
7.294	7.294	6.428	341.952		0.02 × 10^18^	0.0027
7.243	7.243	6.405	335.964	–1.8	1.02 × 10^18^	0.134

a Estimated from the SRIM2013 simulation of displacements produced by an ^241^Am alpha-decay (*i.e.*, an alpha-particle of 5.486 MeV and a ^237^Np recoil nucleus of 92 keV) in AmVO_3_ and AmVO_4_ yielding dose multiplication factors, *d*, from dose to displacement per atom of 1700 and 1800, respectively.

**Figure 4:**
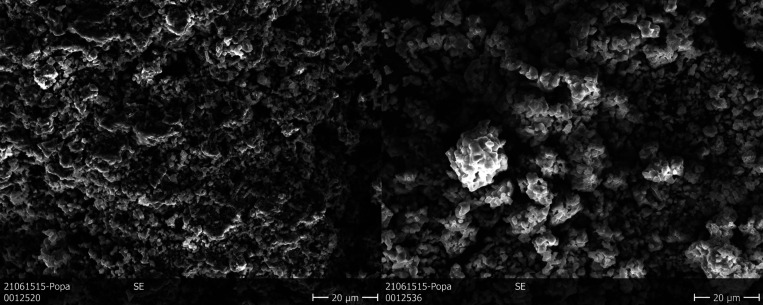
SE images of the microstructure of AmVO_3_ (left) and AmVO_4_ (right) recorded at 660 days after synthesis (5.1 × 10^18^ α decay events/g).

The SE micrographs presented in Figure 4 were recorded about 2 years after the synthesis. Even if the specimens had a relatively high level of self-irradiation at the time of the measurements, it can be observed that the microstructures are typical for solid state reactions, confirming the self-homogenization of the reaction mixture during heat treatment.

**Figure 5:**
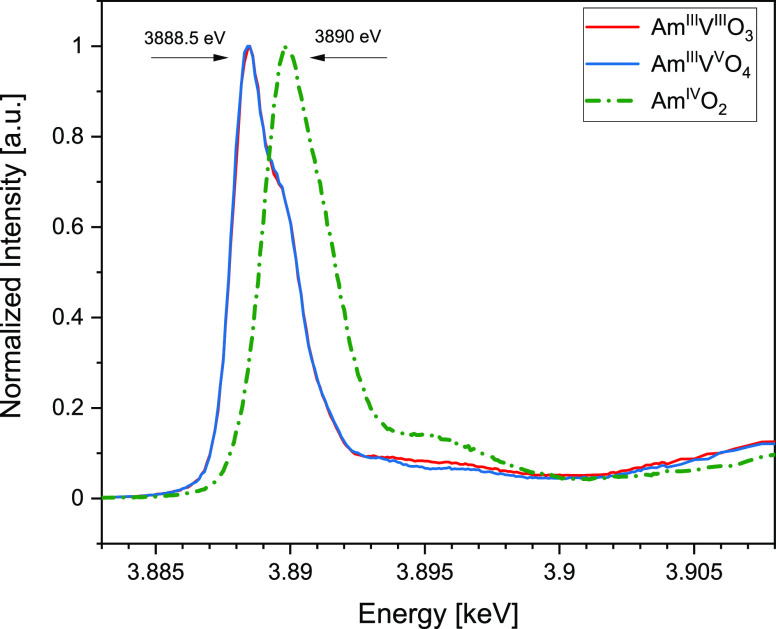
HR-XANES spectra acquired at the Am M_5_ edge for AmO_2_ (green, dash-dotted), AmVO_3_ (red, solid, 4.7 × 10^18^ α/g), and AmVO_4_ (blue, solid, 4.7 × 10^18^ α/g) materials. The maximum of the white line position is indicated for Am^III^ and Am ^IV^ compounds, recorded at 3888.5 and 3890 eV, respectively.

**Figure 6:**
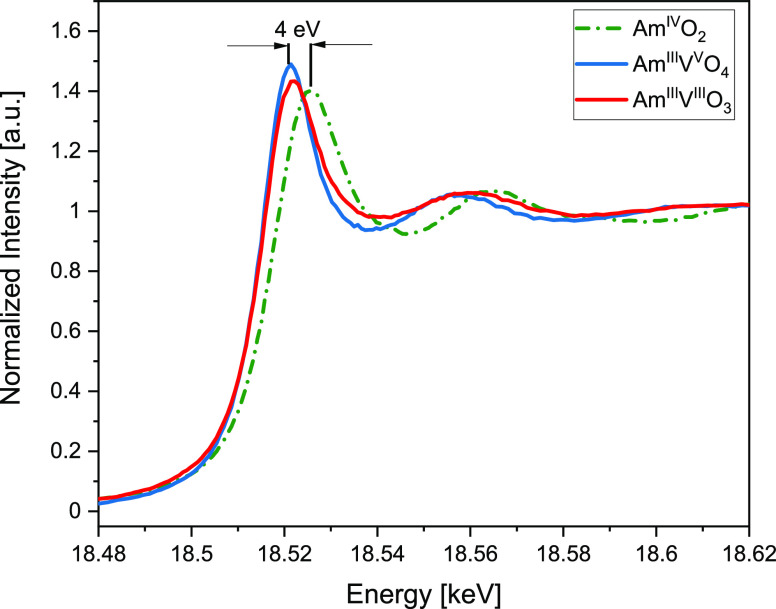
XANES spectra acquired at the Am L_3_ edge for AmO_2_ (green, dash-dotted), AmVO_3_ (red, solid, 4.7 × 10^18^ α/g), and AmVO_4_ (blue, solid, 4.7 × 10^18^ α/g) materials. The shift of the WL maximum of 4 eV is consistent with the chemical shift between Am^III^ and Am^IV^.

**Figure 7:**
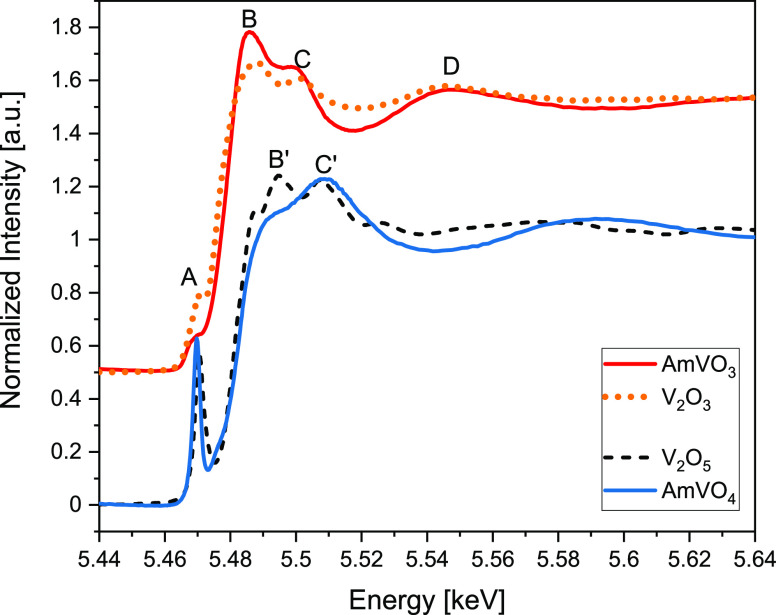
XANES spectra acquired at the V K edge for AmVO_3_ (red, solid, 5.6 × 10^18^ α/g) and AmVO_4_ (blue, solid, 5.6 × 10^18^ α/g) along with V^III^ (orange, dotted) and V^V^ (black, dashed) references.

To assess the oxidation state of both americium and vanadium in the AmVO_3_ and AmVO_4_, XANES spectroscopy characterization was performed. Am M_5_ edge HR-XANES, Am L_3_ edge XANES, and V K edge XANES spectra were collected. The HR-XANES data at the Am M_5_ edge reveals that Am atoms have an Am^III^ oxidation state for both AmVO_3_ and AmVO_4_ compounds. The energy position of the main absorption resonance (white line, WL) maximum in the AmVO_3_ and AmVO_4_ spectra (Figure 5, blue and red solid lines) is located at about 3888.5 eV that is characteristic of the main absorption intensity of Am^III^, consistent with previously reported data.^14,15^ Note that a very limited number of Am M_5_ data are published to date. To be able to compare to published spectroscopic data on compounds containing Am^III^, Am L_3_ edge XANES experiments were also performed prior to collecting the V K-edge data. The AmVO_3_, AmVO_4_, and AmO_2_ XANES spectra were obtained following background subtraction by fitting a linear polynomial to the pre-edge region of the absorption spectrum and normalized at the maximum (WL) intensity for Am M_5_ (Figure 5) and at the high energy range for Am L_3_ and V K edge data (Figures 6 and 7). The maxima of the WLs located at Am^III^VO_3_ (18,521 eV eV), Am^III^VO_4_ (18,521 eV), and Am^IV^O_2_ (18,525 eV) are similar to Am^III^_2_O_3_ and Am^IV^O_2_ reported elsewhere.^22^ The difference in the tail feature around 18,533 eV as well as smearing of the shape-resonance located at about 18,556 eV for AmVO_3_ as compared to AmVO_4_ suggests a different level of amorphization. Therefore, even though at this level of self-irradiation, both AmVO_3_ and AmVO_4_ are lacking a long-range order as observed by XRD, the degree of amorphization is somewhat different as revealed by Raman data (see below) and suggests that amorphization affects less the local structure in AmVO_4_ as compared to AmVO_3_.

To directly compare the electronic structure of vanadium, the background-subtracted and normalized V K-edge XANES spectra for AmVO_3_ and AmVO_4_ (blue and red solid lines, respectively) as well as reference V_2_O_3_ and V_2_O_5_ compounds (orange dotted and black dashed lines, respectively) are shown in Figure 7. As expected, for AmVO_3_, vanadium has a V^III^ oxidation state since all spectral features (A–D) match the spectral features of the V_2_O_3_ reference spectrum. The reduced intensity of the pre-edge feature A in AmVO_3_ compared to V_2_O_3_ may indicate changes in the electronic structure of vanadium as a result of AmVO_3_ amorphization due to self-irradiation effects as well as the influence of Am-presence in the resulting AmVO_3_ after reaction of AmO_2_ and V_2_O_5_ at 1250 °C, which differs from the pure V_2_O_5_ structure. The V K-edge XANES spectrum of AmVO_4_ exhibits a strong pre-edge feature (A) characteristic to V^V^ that is well known to be due to a formally forbidden (very weak) 1s → 3d electronic transition, which is dipole-allowed when the local O_h_ symmetry is distorted.^23−25^ Features B and B′ seem to be smeared out for AmVO_4_, indicating a change of the VO_5_ square-pyramidal polyhedral configuration in V_2_O_5_ to tetrahedral in AmVO_4_. The findings are consistent with previously reported data by Benzi *et al.*(26) for V_2_O_5_ and palenzonaite (natural V^V^) compounds. In that study, feature C′ in the tetrahedral geometry is located at about 5510 eV, matching the position of the shape-resonance C′ observed for AmVO_4_.

In conclusion, all the oxidation states probed through XANES and HR-XANES measurement are in perfect agreement with the expectations based on the initial crystallographic structure of the two materials. The effect of self-irradiation and amorphization has therefore no impact on their oxidation states.

**Figure 8:**
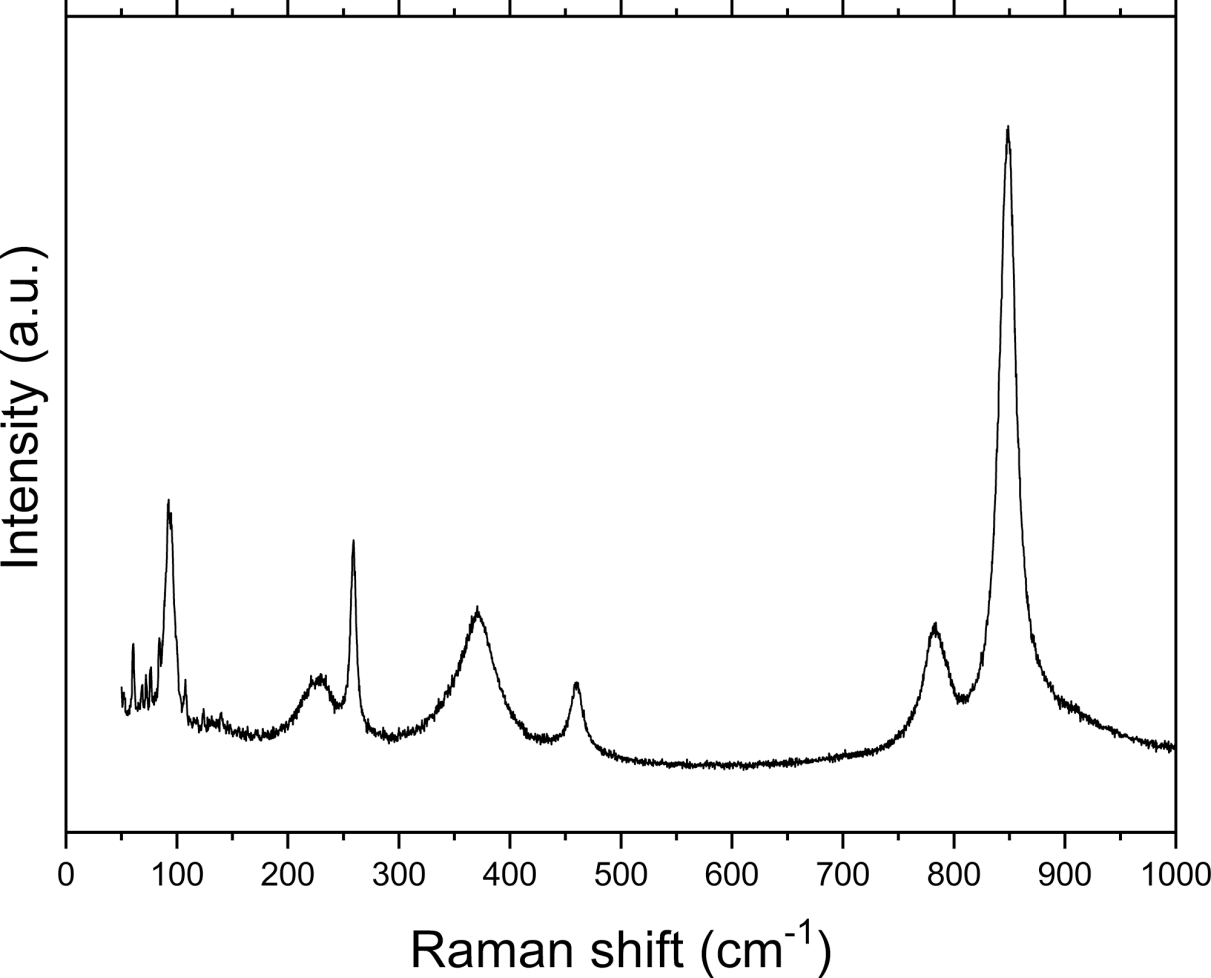
Room-temperature Raman spectrum of AmVO_4_ recorded at 450 days after synthesis (3.3 × 10^18^ α decay events/g).

The Raman spectrum of the aged AmVO_4_ (Figure 8) suggests that the VO_4_ tetrahedra in the zircon-like structure are intact despite the amorphization of AmVO_4_ at this level of self-irradiation found by XRD. The five clearly identifiable internal modes of the [V–O_4_] tetrahedron, ν_1_(A_1g_), ν_3_(B_1g_,E_g_), ν_4_ (B_1g_), ν_2_(A_1g_), and ν_2_ (B_2g_), were found at very similar wavenumbers as for the lanthanide *Ln*VO_4_ zircon compounds,^27^ as shown in Table 5, though somewhat lower than the orthovanadate of neodymium, whose ionic radius is closest to trivalent Am^III^. The two clearly identifiable low-frequency external modes, which reflect the motion between the [V–O_4_] tetrahedron and the Am^III^ ion, were observed, of which the lowest is the T(B_1g_) and the highest is assigned to the R(E_g_). The spectrum also shows a very weak mode at the position of one of the T(E_g_) modes. The T(B_1g_) mode is substantially lower than that found for the lanthanide *Ln*VO_4_ compounds.^27^ This can be explained by the observation by Moura *et al.*(28) that the T(B_1g_) mode in the Tb(V_1–*x*_P_*x*_)O_4_ solid solution shows a different broadening, indicating that it is related primarily to the Tb motion on the [Tb–O_8_] sublattice. The strong shift observed here thus supports that it is due to the mass effect between the actinide and lanthanide series.

**Table 5:** Raman Modes of AmVO_4_ (in cm^–1^) Compared to Some Lanthanide Analogues^a^

	AmVO_4_	LaVO_4_	CeVO_4_	NdVO_4_	SmVO_4_
	this work	Cheng *et al.*(31)	Panchal *et al.*(30)	Nguyen *et al.*(32)	Santos *et al.*(27)
ν_1_(A_1g_)	850	859.0	864.3	871	877
ν_3_(E_g_)	785	796.7	801.3	795	816
ν_3_(B_1g_)	785	783.3	789.1	808	816
ν_4_ (B_1g_)	457	462.3	468.9	472	477
ν_2_(A_1g_)				381	380
ν_4_ (E_g_)	370	376.6	381.1		
ν_2_ (B_2g_)	255	262.9	261.9	375	
T(B_1g_)					261
R(E_g_)	225	228.7	234.1	237	240
T(E_g_)	[140]	141.8			
T(B_1g_)	95	119.7	124.4	123	123
T(E_g_)		109.5		113	112

a We follow the band assignment for CeVO_4_ by Panchal *et al.*(30) that was supported by density functional theory (DFT) calculations.

The shifts of the modes in AmVO_4_ compared to the *Ln*VO_4_ series are very similar to AmPO_4_^5^ and *Ln*PO_4_.^29^ However, the strong broadening of the modes after aging that was observed for AmPO_4_ did not occur. This may be explained by recrystallization of the amorphous AmVO_4_ phase resulting from heating by the incident Raman laser. This would suggest relatively low critical amorphization temperatures, in line with the observations for the monazite and zircon *Ln*PO_4_ analogues.^29^

Surprisingly, the Raman spectrum of the aged AmVO_3_ sample was identical to that of AmVO_4_, suggesting that oxidation took place. We exclude that the oxidation is related to the damage build-up since it is not observed in the XRD and XANES results at similar dose. We therefore conclude that it is triggered by heating the laser during the Raman measurement.

### 3.3 Closer Look at the α Self-Irradiation Mechanism in AmVO_4_

Our results for AmVO_4_ amorphization derived from XRD analysis differ significantly from the observations in the study of Goubard *et al.*,^20^ who studied the radiation damage build-up in AmVO_4_ during a much longer period of 5 years (14 × 10^18^ α/g). They reported that the structure remained at least partially crystalline during this period and became predominantly amorphous after 5 years, which is about 10 times slower than in our work. Moreover, Goubard *et al.*(20) did not observe a volume contraction under α self-irradiation like in the present study, but a moderate lattice expansion with a volume increase to a maximum of about Δ*V*/*V*_0_ = 1.2%. At a comparable dose to our study (1 × 10^18^ α/g), they found Δ*V*/*V*_0_ = 0.15%.

The reasons for the discrepancies with the work of Goubard *et al.*(20) remain unclear, and one can only hypothesize the following:

- A different content of neptunium (daughter element of americium) in the initial material. Note that Goubard *et al.*(20) did not give details about the purity of the used Am. The exact quantity of Np that could be incorporated in AmVO_4_ is not known, but we observed NpO_2_ as the separate phase in this work. Moreover, uptake of Np^IV^ in the structure would require charge compensation, for example, by V^III^, which was not observed by XANES in our work.
- The higher synthesis temperature in the current work (1000 °C vs 600 °C), which could have resulted in a more crystalline material and thus less initial disorder. As a result, a potential time span of contraction could have been absent or short in the study by Goubard *et al.*(20) It should be noted that their first data point at 0.7 × 10^18^ α/g is anomalous in their Δ*V*/*V*_0_ vs dose curve and indicates a minimal expansion (Δ*V*/*V*_0_ = 0.03%) after approximately 90 days.
- The difference in production scale (hundreds of milligrams here versus 1 mg by Goubard *et al.*(20)) and more probable methods (synthesis temperature and duration) could have resulted in substantially different crystallite sizes, powder morphologies, and densities, thus potentially affecting the impact of the alpha decay through annihilation of defects, in particular via diffusion to sinks (grain boundaries).^33^
- Radiogenic helium can interact with defects (vacancies), but the effect of this will depend on the microstructure and crystallinity. The larger the grain size, the larger the helium fraction retained in the lattice, affecting the defect recombination kinetics. Similarly, the initial disorder (crystallinity) will affect the helium retention. Unfortunately, information on the microstructure of the material synthesized by Goubard *et al.*(20) is missing.

The first three hypotheses could explain the slight difference of the initial lattice parameters (see Table 2), but not the different kinetics. Although the damage kinetics could be affected by size and geometry of samples analyzed in the XRD instruments, the discrepancy is huge. The last two hypotheses could explain the different recombination kinetics, but remain speculative, in the absence of comparative microstructural data.

Our observations for zircon-type AmVO_4_ are different from our results for the monazite-type AmPO_4_.^5^ The zircon structure is closely related to the monazite *LnM*O_4_ structure, both made of isolated tetrahedra of V or P, connected by trivalent metal ions in eight or nine coordinations, respectively. Under the influence of temperature and/or pressure, a transition can take place.^30,31^ For AmPO_4_, a significant expansion of the unit cell was observed (Δ*V*/*V*_0_ = 1.8% at 0.6 × 10^18^ α/g), whereas the AmVO_4_ cell contracts with irradiation time. However, the dose at which the transition to a fully amorphous phase takes place is close in both studies, slightly higher for AmVO_4_ compared to AmPO_4_, which is in line with the observation of Meldrum *et al.*(29) that the critical amorphization temperature is slightly higher for zircon-type *Ln*PO_4_ compounds as well.

These contrasting observations are not easy to combine. Volume contraction due to α self-irradiation is known for Am_2_Zr_2_O_7_ pyrochlore^34^ and was attributed to the stability of the [Zr–O_6_] octahedra and their rotation in response to the increase in disorder around americium. Although Am_2_Zr_2_O_7_ has a close-packed pyrochlore structure, which is very different from the open oxyanionic structures of zircon and monazite, this explanation may help understand. The volume in the *Ln*VO_4_ series is discontinuous, with the zircon (xenotime) structure (eight-coordinated *M*^III^) being larger than the monazite (nine coordination),^35^ similar to the *Ln*PO_4_ series.^36^ So, in case the radiation damage in zircon-type AmVO_4_ affects predominantly the americium coordination sphere leading to a higher coordination with a concomitant rotation of the [V–O_4_] tetrahedra, the volume will decrease, as we observed here. In AmPO_4_, the Am ions are already nine-fold coordinated, resulting in prompt expansion of the lattice with increasing dose. Of course, also differences in the radiation resistance of the [V–O_4_] and [P–O_4_] tetrahedral entities may play a role.

## 4 Conclusions

Two americium vanadates were produced and characterized in terms of crystal structure, cation oxidation states, and chemical, thermal, and radiation stability. The XRD and XANES measurements demonstrate that americium stays in the oxidation state III in the material whatever the redox condition, while vanadium shows the oxidation state III in the perovskite-like AmVO_3_ or V in the zircon-like AmVO_4_.

After an accumulation of a dose of 10^18^ α/g, the close-packed AmVO_3_ shows a volume expansion of 4.2% while the low density oxyanionic AmVO_4_ contracts with 1.8% in volume (in contrast with other literature reports^20^). With a fast amorphization behavior, large volume variation, and interchangeable structure as a function of the presence of the oxygen atmosphere and temperature, americium vanadates do not appear to be good candidates as Am forms for radioisotope power systems. Moreover, ^237^Np (the decay product of ^241^Am) tends to segregate as a fluorite fcc secondary phase, creating interfaces and inducing stress in the material. Comparing the existing data on Am-containing ceramics with fluorite,^3^ pyrochlore,^34^ zircon, monazite,^5^ and perovskite^4^ structures, the (Am,U)O_2_^2,3^ fluorite solid solution seems currently to be the most suitable form for space applications.

## Supporting Information Available

Thermogravimetric behavior of AmVO_3_ and AmVO_4_; overview of the AmVO_3_ and AmVO_4_ specimens measured by SEM and EDX; XRD diffraction pattern of AmVO_3_ after TGA measurement; Np L_3_ XANES spectra of the AmVO_3_ and AmVO_4_; discussion on the difference in the experimental energy resolution leading to an energy shift of the main maxima of the An M_4,5_ edge HR-XANES spectra; discussion on the neptunium dioxide residual phase from XRD data (PDF)

## Notes

J.-F.V.: Conceptualization, formal analysis, investigation, methodology, validation, and writing (original draft; review and editing); T.W.: Investigation and writing (review and editing); N.P.: Formal analysis, investigation, methodology, validation, and writing (original draft; review and editing); T.V.: Formal analysis, funding acquisition, project administration, validation, and writing (review and editing); J.-Y.C. and D.B.: Investigation; D.F.: Project administration, resources, and writing (review and editing); R.J.M.K.: Project administration, resources, supervision, and writing (review and editing); K.P.: Conceptualization, investigation, methodology, supervision, and writing (original draft; review and editing).
