# Supplementary material for: Synthesis, Characterization, and Stability of Two Americium Vanadates, AmVO3 and AmVO4
Source: Inorg Chem. 2023 Jun 5;62(24):9350–9. doi: 10.1021/acs.inorgchem.3c00251 (PMC10283022; doi:10.1021/acs.inorgchem.3c00251)
Supplement: Supplementary file 1 — ic3c00251_si_001.pdf [file ic3c00251_si_001.pdf]

Synthesis, characterization and stability of two americium vanadates, $AmVO_3$ and $AmVO_4$

Supplementary information

Jean-François Vigier,*[,1] Thierry Wiss,[1] Natalia Palina,[2] Tonya Vitova,[2] Jean-Yves Colle,[1] Daniel Bouëxière,[1] Daniel Freis,[1] Rudy J.M. Konings,[1] Karin Popa,**[,1]

[1] *European Commission, Joint Research Centre (JRC), Karlsruhe, Germany*

[2] *Institute for Nuclear Waste Disposal (INE), Karlsruhe Institute of Technology, P.O. 3640, D-76021 Karlsruhe, Germany*

* *Corresponding author, e-mail: jean-francois.vigier@ec.europa.eu*

** *Corresponding author, e-mail: karin.popa@ec.europa.eu*

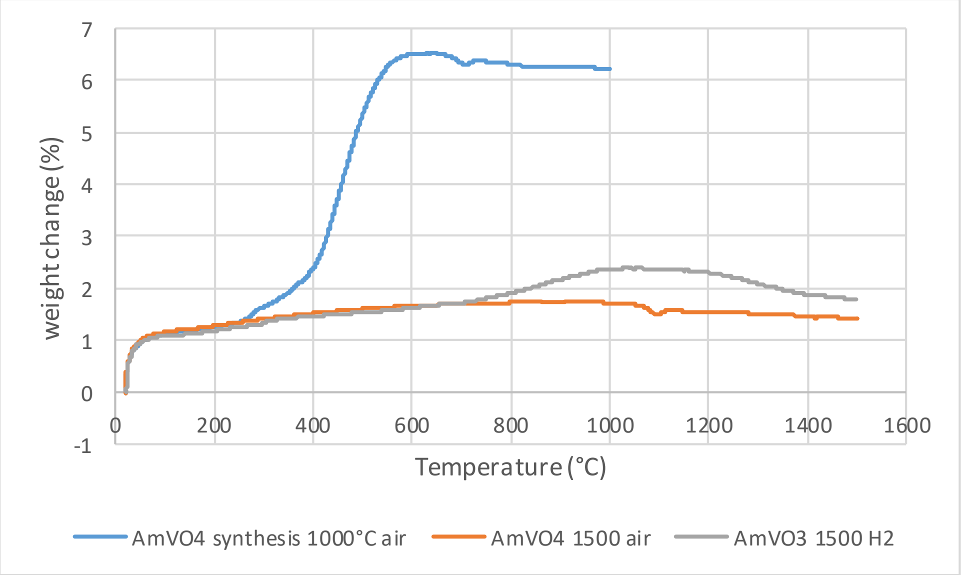

Figure S1 Thermogravimetric analysis (TGA) profiles for the conversion of $AmVO_3$ into $AmVO_4$ (air), $AmVO_4$ (air) and $AmVO_3$ (Ar/H_2)

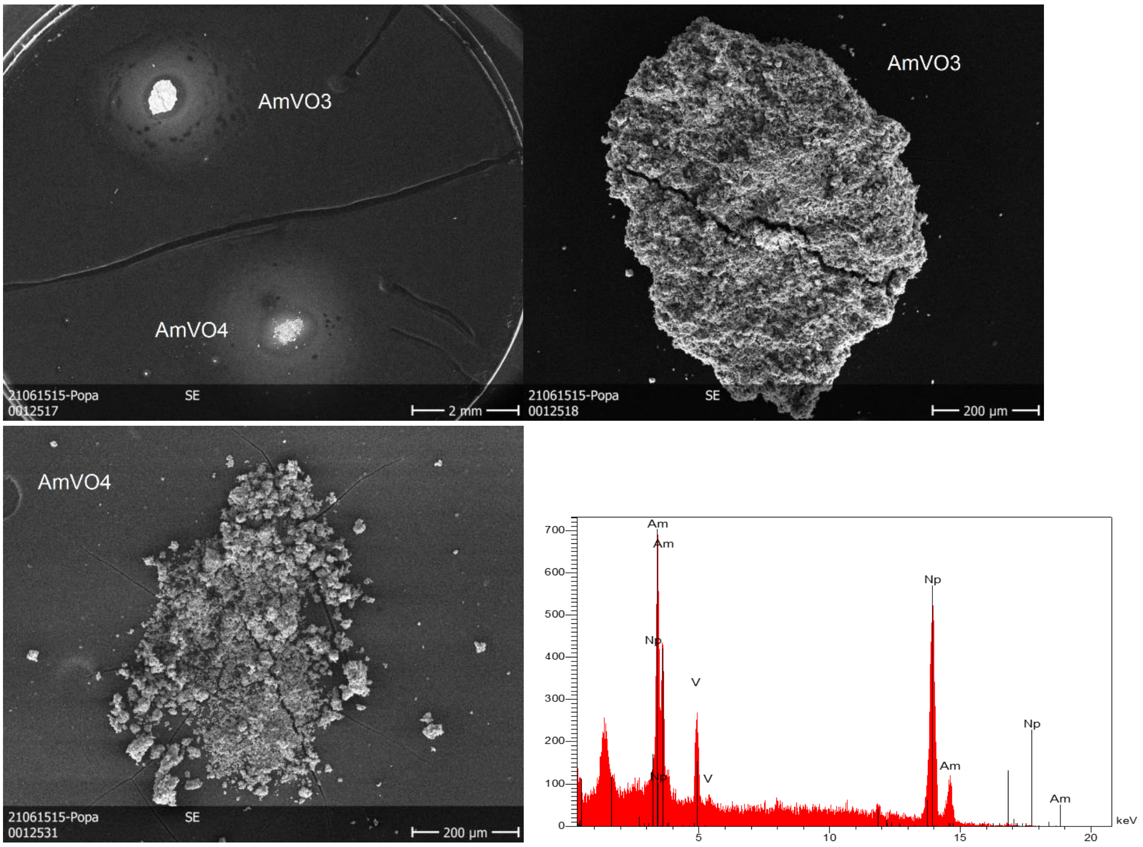

Figure S2 Overview of the $AmVO_3$ and $AmVO_4$ specimens measured by SEM; note the important mechanical damage induced by simple pressing of the aged $AmVO_4$ specimen on the stub. Bottom right: typical EDX spectrum of $AmVO_3$ indicating the presence of the Np in addition to Am; semi-quantitative quantification gives an appropriate composition of V/(Am+Np) ≈ 50/50

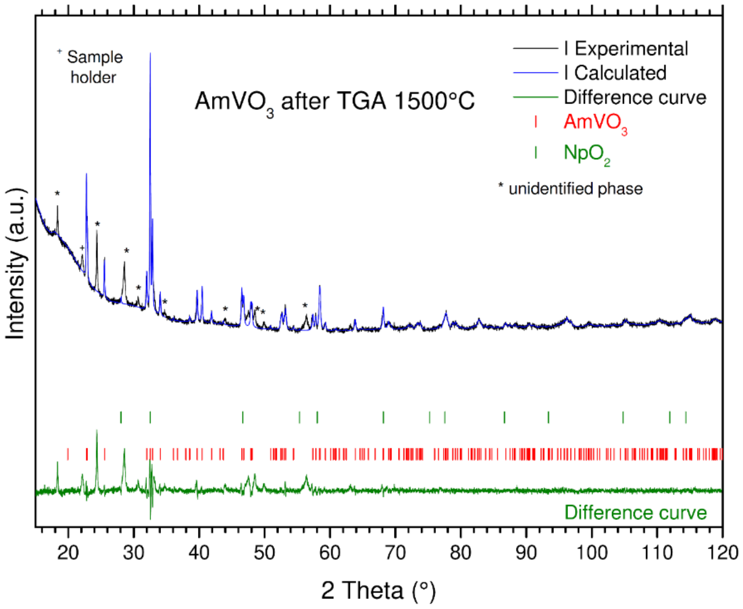

Figure S3 XRD diffractogram of $AmVO_3$ after TGA indicating the presence of an unidentified crystalline phase

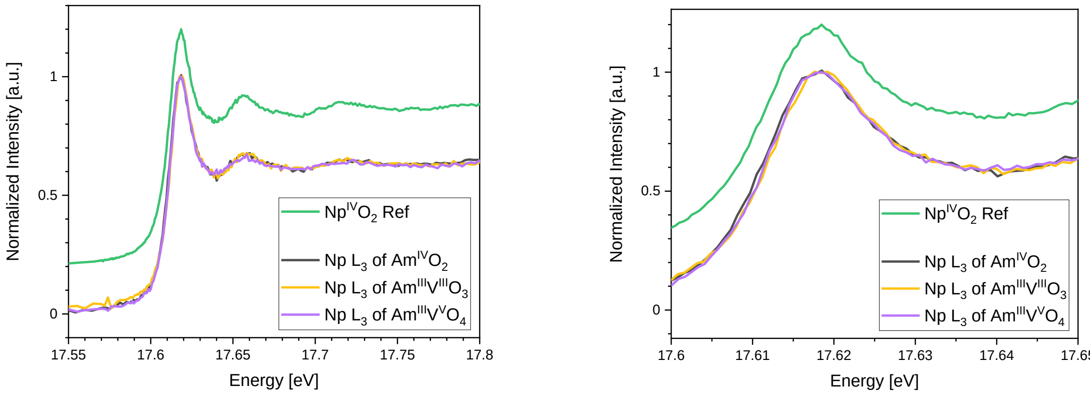

Figure S4 (left) Np L_3 XANES spectra of the NpO_2 (green) reference and $AmVO_3$ (yellow), $AmVO_4$ (lilac) and AmO_2 (black) compounds, respectively. (right) Zoom around the white line position (17618.5 eV).

Np L_3 edge (tabulated energy position of L_3 absorption edge is 17610 eV) measurements were performed at the INE-Beamline [Rothe 2012] of the KIT Light Source, Karlsruhe, Germany. Two Ge(422) crystals were mounted in the double-crystal monochromator (DCM) for Np L_3 measurements. The beam was focused on a ~0.5 mm by 0.5 mm spot on the sample. Zr- foils (energy position of K absorption edge is 17998 eV) were used for energy calibration for the Np L_3 edge XANES. The XANES spectra were measured in fluorescence mode with a step size of 0.8 eV from -30 eV to +30 eV from the white line (WL) of the Np L_3 edge. Measurements were performed in air and no radiation damage was observed during the measurements.

Discussion on the difference in the experimental energy resolution leading to energy shift of the main maxima of the An $M_{4,5}$ edge HR-XANES spectra

At present, the HR-XANES technique is renowned for its capability to access electronic information on the materials, not accessible otherwise, as the enhanced spectral features of HR-XANES provide an advantage over conventional XANES due to better energy resolution.

For the discussion below, we will refer to the Supplementary Figure 2 in Vitova *et al.* [Vitova 2017], https://www.nature.com/articles/ncomms16053#MOESM361: Pu M_5 edge HR-XANES/XANES and 3d4f RIXS spectra of PuO_2^{2+}. (a-b) Pu M_5 edge HR-XANES/XANES spectra of PuO_2^{2+} obtained by integrating regions with variable emission energy widths (green: 0.05 eV, pink: 0.25 eV, cyan: 1 eV, blue: 5 eV, orange: 10 eV) across the 3d4f RIXS map of PuO_2^{2+}. These are line A (3351.6 eV) (a) and line B (3350 eV) (b) centered regions; (c) as an example the line B centered regions of the 3d4f RIXS map. The XANES spectrum in (b) obtained integrating a region with emission energy width of 10 eV corresponds to a Pu M_5 edge XANES spectrum measured in conventional fluorescence mode.

It is to consider that the HR-XANES spectra are in principle cuts across core-to-core Resonant Inelastic X-ray Scattering (RIXS) maps. Due to the long measurement time, often not a complete CC-RIXS map is measured but a HR-XANES spectrum. This is carried out by positioning the X-ray emission spectrometer at the maximum of the normal emission line marked with line B in the example in Fig. S5 and scanning only the excitation energy. Depending on the experimental energy resolution of the X-ray emission spectrometer, the region integrated along the emission energy scare is very small or

very large (cf. **Fig. c**). The former is a case for good and the latter for bad spectrometer energy resolution. For certain actinide materials, the main absorption resonance is shifted to higher energy with respect to the energy position of the main maximum of the normal emission line (cf. **Fig. c**). This results in an energy shift of the main maximum of the HR-XANES spectra at the $M_{4,5}$ edge to higher excitation energy when the spectrometer energy resolution decreases. This is demonstrated in **Fig. b**.

Note that the discrepancy in the WL position of AmO_2 in the work published by Epifano *et al.* [Epifano 2019] and the current work is due to the lower spectrometer energy resolution in the work of Epifano *et al.* originating from the larger beam size of 500 micrometer in diameter on the sample compared to 200 micrometer vertical size in the current work. Since the vertical Rowland circle focusing spectrometer was applied in both cases, the vertical beam size is important for the spectrometer energy resolution.

As HR-XANES spectrum is reflecting electronic structure of the studied material, the shape is also affected by the elemental configuration. In the work of Epifano *et al.* [Epifano 2019], AmO_2 had been produced using UMACS process that is known to result in AmO_2 with several percent of impurities, including lanthanides. As a result, the electronic structure of Am(IV) might experience minor changes. That should not be a case for the sample preparation used in the current work. Presence of a specific impurities, as well as discrepancy in experimental resolution can affect HR-XANES also trackable as a shift of the WL position.

Extended discussion on the neptunium dioxide residual phase from XRD data

Some additional discussion on the results concerning the dioxide phase (NpO_2 possibly containing Am) fractions and their lattice parameters can be attempted. However, due to the small quantities of these phases in the two materials and their overlap with the main vanadate phase, the reliability of the results is difficult to evaluate and is probably low, the interpretation should then be done with a lot of care.

Concerning the $AmVO_3$ sample, the lattice parameter of the oxide phase is very large compared to that of $AmO_{2.00}$ and $NpO_{2.00}$. This can be easily explained by the presence of a significant amount of americium remaining in the oxide phase at this stage and having a trivalent oxidation state. Indeed, americium (III) has a very large ionic radii compared with Am(IV) or Np(IV) [Shannon 1976] and with such a large lattice parameter one can expect that americium (III) represents still about half of the actinide composition in the oxide. Furthermore, it seems that the lattice parameter of the oxide phase is increasing over time with an overall increase in fair agreement with reported values of other dioxide under alpha self-irradiation, which tends to confirm that significant amount of americium is present in the dioxide. Finally, diffraction peaks of the oxide phase are relatively intense and asymmetric, which is commonly observed for strongly sub-stoichiometric dioxide MO_{2-x}.

On the other hand, despite this probable presence of a significant amount of americium in the oxide phase, it is anyway expected that neptunium segregated in this phase with a Np/(Am+Np) ratio much higher than the initial 7% since the main oxide phase after reductive sintering would be the hexagonal sesquioxide M_2O_3 and not a fluorite dioxide MO_{2-x}. By comparison with previous reductive sintering of americium oxide only, containing the same amount of neptunium [Vigier 2018, SI], we expect the oxide phase to contain at least 25% of neptunium.

After oxidation into $AmVO_4$, the lattice parameter of the oxide phase significantly decreases. Here again, the initial value obtained should be consider with care since only one diffraction peak with

significant intensity does not overlap with vanadate phase. The values seem anyway in very good agreement with pure neptunium dioxide, $NpO_{2.00}$. Furthermore, despite a slight scattering of the values, the lattice parameter of the oxide phase does not seem to change significantly over time, which would support the absence of americium in this phase. The change of the fraction of oxide phase during oxidation (from 3% to 1% of the material) can be then a migration of americium from oxide to vanadate phase during oxidation or a combination of this first phenomena with a potential different level of solubility of neptunium in $AmVO_3$ and $AmVO_4$.

Finally, as expected, one can see that NpO_2 concentration obtained by Rietveld refinement (ratio of NpO_2 over crystalline phases only) increase with amorphization process. Considering that the material contains initially only crystalline phases, and that the NpO_2 phase stays crystalline under self-irradiation, we can use the change of this proportion to evaluate the amount of amorphous vanadate for self-irradiated materials. The results are presented in **Table S1**.

Table S1: Lattice parameters and phase proportion of NpO_2 phase according to Rietveld refinement, and deduced amount of amorphous vanadate in the materials.

	alpha/g	*a* NpO_2, Å	*x* NpO_2, %	estimation of amorphous vanadate according to change of *x* NpO_2	Amorphous vanadate/ Vanadate total	Reference
$AmVO_3$	1E+16	5.494(1)	2.7	0%	0.0%	
	1.0E+18	5.504(1)	4.8	43.8%	45.0%	
	1.6E+18	5.503(1)	17	84.2%	86.6%	
$AmVO_4$	1E+16	5.431(3)	0.69	0.0%	0.0%	
	1.0E+18	5.439(1)	4.4	84.3%	84.9%	
	1.6E+18	5.435(1)	100	99.3%	100.0%	
AmO_2		5.377				[Lebreton 2012]
NpO_2		5.434				[Martel 2014]

References

[Epifano 2019] Epifano, E.; Naji, M.; Manara, D.; Scheinost, A. C.; Hennig, C.; Lechelle, J.; Konings, R. J. M.; Guéneau, C.; Prieur, D.; Vitova, T.; Dardenne, K.; Rothe, J.; Martin, P. M. Extreme multi-valence states in mixed actinide oxides. *Commun. Chem.* **2019**, *2*, 59

[Lebreton 2012] Lebreton, F.; Belin, R. C.; Prieur, D.; Delahaye, T.; Blanchart, P. In situ study of the solid-state formation of $U_{(1-x)}Am_{(x)}O_{(2\pm\delta)}$ solid solution. *Inorg. Chem.* **2012**, *51*, 9369-9375

[Martel 2014] Martel, L.; Vigier, J.-F.; Prieur, D.; Nourry, S.; Guiot, A.; Dardenne, K.; Boshoven, J.; Somers, J.; Structural investigation of uranium-neptunium mixed oxides uding XRD, XANES, and ^{17}O MAS NMR. *J. Phys. Chem. C* **2014**, *118*, 27640-27647

[Rothe 2012] Rothe, J.; Butorin, S.; Dardenne, K.; Denecke, M.A.; Kienzler, B.; Löble, M.; Metz, V.; Seibert, A.; Steppert, M.; Vitova, T.; Walther, O. and Geckeis, H. The INE-Beamline for actinide science at ANKA. *Rev. Sci. Instrum.* **2012**, 83, 043105

[Shannon 1976] Shannon, R. D. Revised effective ionic radii and systematic studies of interatomic distances in halides and chalcogenides. *Acta Cryst.* **1976**, *A32*, 751-767

[Vigier 2018] Vigier, J.-F.; Freis, D.; Pöml, P.; Prieur, D.; Lajarge, P.; Gardeur, S.; Guiot, A.; Bouëxière, D.; Konings, R. J. M. Optimisation of uranium-doped americium oxide synthesis for space applications. *Inorg. Chem.* **2018**, *57*, 4317-4327

[Vitova 2017] Vitova, T.; Pidchenko, I.; Fellhauer, D.; Bagus, P. S.; Joly, Y.; Pruessmann, T.; Bahl, S.; Gonzales-Robles, E.; Rothe, J.; Altmaier, M.; Denecke, M. A.; Geckeis, H. The role of the 5f orbitals of early actinides in chemical bonding. *Nature Commun.* **2017**, *8*, 16053
